# Hypercalcemia in children with APL caused by interactions between voriconazole and all-trans retinoic acid: A case report and literature review

**DOI:** 10.1097/MD.0000000000041426

**Published:** 2025-02-07

**Authors:** Namei Wu, Zhihang Lin, Shuquan Zhuang, Shuifa Wu, Zhiming Cai, Xiaofang Wang

**Affiliations:** a Department of Pharmacy, Fujian Medical University Affiliated First Quanzhou Hospital, Quanzhou, China; b Department of Pediatric, Fujian Medical University Affiliated First Quanzhou Hospital, Quanzhou, China.

**Keywords:** acute promyelocytic leukemia, all-trans retinoic acid, hypercalcemia, voriconazole

## Abstract

**Rationale:**

All-trans retinoic acid (ATRA) and voriconazole (VRZ) are pivotal drugs for the treatment of acute promyelocytic leukemia (APL) and invasive fungal infections, respectively. When ATRA is co-administered with VRZ, clinically significant drug interactions may occur due to alterations in drug metabolism and clearance.

**Patient concerns:**

We report a case of hypercalcemia caused by the interaction between ATRA and VRZ in a child with APL.

**Diagnoses and interventions:**

A 14-year-old boy received arsenictrioxide (ATO) for APL and VRZ for invasive fungal infections, followed by planned maintenance therapy with ATRA monotherapy and combination of ATRA and ATO. He experienced no adverse reactions during the concurrent use of ATO and VRZ, while on the 12th day of combined ATRA and VRZ administration, his blood calcium levels significantly increased, accompanied by a series of symptoms. Following the discontinuation of VRZ and continuation of ATRA monotherapy, and subsequent maintenance chemotherapy with ATRA and ATO, his blood calcium levels decreased and remained within the normal range.

**Outcomes:**

We reviewed the published literature and excluded primary hyperparathyroidism or ectopic parathyroid hormone secretion as the cause of hypercalcemia in the child. He did not use other cytochrome inhibitors that may affect ATRA metabolism other than VRZ. Multiple measurements of VRZ trough concentrations ranged from 1.2 to 3 μg/mL. According to the Drug Interaction Probability Scale (5 points) and Naranjo Probability Scale (4 points), the drug interaction between VRZ and ATRA is probable. The hypercalcemia and other clinical manifestations may be caused by the inhibition of ATRA metabolism by VRZ.

**Lessons:**

During clinical use of ATRA, it is necessary to closely monitor the adverse drug interactions such as hypercalcemia and limit the use of drugs that may affect cytochrome P450 enzyme such as VRZ.

## 1. Introduction

Acute promyelocytic leukemia (APL) is an unique subtype of acute myeloid leukemia characterized by a balanced reciprocal translocation between chromosomes 15 and 17, leading to the oncogenic fusion protein promyelocytic leukemia-retinoic acid receptor α (PML-RARα)^[1]^ by targeting PML-RARα fusion protein, induction therapy based on all-trans retinoic acid (ATRA) and arsenictrioxide (ATO) resulted in remission and near-100% survival.^[2]^ Due to long-term chemotherapy, hematopoietic stem cell transplantation and immunosuppressive therapy, patients with APL are immunocompromised and prone to invasive fungal infections (IFIs). Aspergillus and Candida are the most common pathogens of IFIs in patients with hematological malignancies.^[3]^ Triazole antifungal drugs such as voriconazole (VRZ) are important choices for the treatment of IFIs, and are widely used for the prevention of fungal infection in patients with solid organ transplantation, malignant blood diseases, etc.^[4]^ When ATRA is combined with triazole antifungal agents, drug metabolism and clearance are altered, resulting in clinically significant drug interactions. Here, we report a case of hypercalcemia in children with APL caused by the interaction of ATRA and VRZ.

## 2. Case report

A 14-year-old Chinese boy (weight 50 kg, height 176 cm, body mass index 3.1 kg/m^2^) was diagnosed as APL with positive PML-RARα fusion gene of breakpoint cluster region 1 type and low-risk. ATRA combined with ATO were used for induction chemotherapy. Before this admission, he had completed 3 rounds of consolidation chemotherapy, 1 round of maintenance chemotherapy, and had started second round of maintenance chemotherapy 1 month earlier. The boy was admitted due to myelosuppression, neutropenia and infection after chemotherapy, and received cefoperazone/sulbactam from day 1 to day 17 (Day1–17) of hospitalization for antiinfection and ATO (Day1–26) for maintenance chemotherapy. Invasive pulmonary fungal infection was considered since computed tomography indicated bilateral lung inflammation, and bronchial alveolar lavage fluid 1-3-β-d-glucan test and galactomannan antigen test were positive. VRZ 200 mg q12h was administered on Day17 and adjusted to 300 mg q12h on Day36. The 28-day ATRA 25 mg/(m^2^·d) maintenance treatment program was started on Day42. The trough concentration of VRZ (C_VRZ_) was 2.0 μg/mL. During Day44 to 48, he experienced vomiting accompanied by paroxysmal headache, which was improved after receiving ondansetron for antiemetic and fluid replacement therapy.

As computed tomography showed no obvious improvement in lung lesions, VRZ 350 mg q12h was sequentially administered orally at Day52. His serum calcium concentration (C_CA_) rose to 4.06 mmol/L on Day53, and vomiting recurred. Immediate fluid replacement therapy for hypercalcemia was administered to promote calcium excretion. Three days later, the vomiting improved, but he newly developed chapped lips and skin, and pain in the right lower limb, which worsened when standing and walking. There was no abnormality in urine routine test, and 24-hour urine quantitative monitoring showed urine volume of 2.97 L/24 h, urine calcium 3.9 mmol/24 h, urine sodium 49 mmol/24 h, and urine phosphorus 4.53 mmol/24 h. Parathyroid hormone (PTH) was 6.00 ng/L. The electrocardiogram showed sinus bradycardia and left ventricular high voltage. The C_VRZ_ was 2 µg/mL on Day56, and VRZ dosage was reduced to 300 mg q12h. On Day60, C_VRZ_ was remeasured at 3.0 µg/mL. The C_CA_ monitored on Day55, 57, 58, and 60 were 3.61, 3.26, 2.41, and 2.87 mmol/L, respectively.

On Day63, the child developed membranous skin peeling with erythema, swollen, desquamation, and pruritus, which was pronounced on the limbs. In addition, he complained of pain in the left lower limb while walking. C_CA_ was 3.45 mmol/L and C_VRZ_ was 1.6 µg/mL on Day64, and C_CA_ was monitored at 3.66 mmol/L on Day65. The skin peeling improved, redness and swelling subsided, but C_CA_ rose to 4.13 mmol/L the following day. After fluid supplement and salmon calcitonin (100 IU q12h from Day66 to Day70) treatment, C_CA_ decreased to 3.14 mmol/L on Day67. VRZ was subsequently discontinued and ATRA chemotherapy was continued. On Day69, C_CA_ decreased to 2.58 mmol/L. His lower limb pain almost relieved, skin membranous peeling, redness completely subsided on Day70, so salmon calcitonin was stopped subsequently. He received adjusted maintenance chemotherapy with ATRA combined with ATO on Day71. The C_CA_ decreased again to 2.34 mmol/L, and which remained within normal range on follow-up monitoring. The main medications and selected laboratory values during hospitalization were shown in Table 1.

**Table 1:**
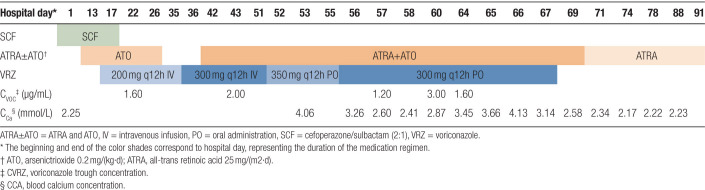
The main medications and selected laboratory values during hospitalization.

## 3. Discussion and literature review

ATRA is metabolized by cytochrome P450 enzymes (CYP450), with CYP2C9, CYP3A4, and CYP2C8 being the most significant ones.^[5]^ Individual differences in the expression of CYP subtypes may influence the pharmacokinetics of ATRA. In addition, concurrent use of drugs that undergo CYP450 metabolism or inhibit CYP450 may interfere with ATRA metabolism, leading to increased plasma concentration of the latter. Significant adverse reactions of ATRA include retinoic acid syndrome, pseudotumor cerebri (PTC), and hypercalcemia,^[6]^ and there are also reports of ATRA causing genital and oral ulcers.^[7,8]^ The toxicity of ATRA is associated with high concentrations, that is, with dose-dependent reversible toxicity. Triazole antifungal drugs, such as fluconazole, itraconazole and VRZ, are mainly metabolized by CYP450, and are strong inhibitors of CYP450. When ATRA is combined with triazole antifungal drugs, changes occur in drug metabolism and elimination, leading to clinically significant drug interactions.^[9,10]^

PTC, a manifestation of ATRA central nervous system toxicity, is characterized by neurological and ocular symptoms as well as signs of increased intracranial pressure, including headache, nausea, papilledema, diplopia, and elevated blood pressure. Fluconazole was shown to inhibit NADPH-dependent CYP450-mediated catabolism of ATRA in a concentration dependent manner.^[11]^ Vanier et al^[12]^ reported a 4-year-old patient with APL who developed PTC during the use of therapeutic doses of ATRA (45 mg/m^2^ per day) and presented with headache, vomiting, and papilledema. Reducing the ATRA dose to 30%, headache symptoms remained. After discontinuation of fluconazole, the patient then tolerated full doses of ATRA. Apparently, PTC appeared to be enhanced by concomitant fluconazole use. Dixon et al^[13]^ reported a case of PTC secondary to inhibition of ATRA metabolism by VRZ in an adult patient. The patient developed blurred vision, hyperopia, dry skin with pruritus after concomitant treatment with ATRA and VRZ, and was subsequently diagnosed with PTC secondary to ATRA toxicity. After discontinuation of ATRA, all symptoms of PTC disappeared.

Hypercalcemia that occurs during concomitant use of triazole antifungals with ATRA has been described several times. Cordoba^[14]^ reported hypercalcemia resulting from an interaction of ATRA with itraconazole in the treatment of APL and successfully treated with zoledronic acid. Although posaconazole is rarely metabolized by CYP, like other triazoles, posaconazole inhibits CYP3A4. Recently, cases of hypercalcemia in patients with APL caused by the interaction between ATRA and posaconazole have also been reported.^[15]^

VRZ is metabolized by CYP2C19, CYP3A4, and CYP2C9, and is also a strong CYP3A4 inhibitor. When used in combination with ATRA, VRZ inhibits ATRA metabolism, thereby increasing the serum concentration of ATRA and enhancing its side effects, leading to an increase in blood calcium. Hashmi et al^[6]^ reported a case of a 9-year-old girl who received ATRA for APL and VRZ for extensive fungal chest infection. During the follow-up period, asymptomatic hypercalcemia was found, and the serum calcium level returned to normal within 6 days after discontinuation of the combination of ATRA and VRZ. Bennett reported a case of a 24-year-old APL patient with a marked increase in serum calcium on 13th day after the restart of ATRA and VRZ. Work-up to rule out other causes of hypercalcemia was negative, including PTH, PTH-related protein, adrenal cortical response to cosyntropin and thyroid function. Omeprazole used during the same period may further exacerbate ATRA inhibition by increasing VRZ levels. Hypercalcemia was treated by cessation of ATRA and intravenous pamidronate.^[16]^

The mechanism of ATRA-induced hypercalcemia is considered to be a direct effect of drugs on osteoclast activity, thereby accelerating mineral absorption, increasing serum interleukin-6 levels, enhancing bone resorption, and increasing the level of PTH-related protein. Another mechanism involves the inhibition of CYP450 during hepatic metabolism, among which CYP2C9 and CYP3A4 subtypes are the most important. Drugs including triazole antifungals modulate these enzymes in an inhibitory manner, enhancing the effect of ATRA on calcium metabolism.^[14,17]^

The other clinical manifestations of this case during the period of hypercalcemia were vomiting, headache, chapped lips and skin, systemic membranous skin desquamation, pruritus, and lower limb pain. Monitoring of renal function, urine routine, 24-hour urinary calcium level, and serum PTH concentration ruled out primary hyperparathyroidism or ectopic PTH secretion as the cause of hypercalcemia. Other than VRZ, the child did not use other CYP inhibitors that may affect ATRA metabolism. C_VRZ_ were in the range of 1.2 to 3.0 µg/mL on several occasions. Since it is still unclear whether hypercalcemia is caused by ATRA itself or specific metabolites, measuring ATRA levels may not be beneficial,^[15,16]^ and ATRA levels are not yet routinely measured in hospitals, we did not monitor ATRA concentrations.

In this case, no adverse effects occurred with the concomitant use of ATO and VRZ between Day17 and Day26. ATRA and VRZ were used simultaneously from Day42, and the serum calcium level increased significantly on Day53, which was 12th day of the combination. On Day67, VRZ was stopped, and ATRA was used alone. After that, ATRA combined with ATO were used for maintain chemotherapy. The serum calcium of the child was reduced and maintained at normal level. According to the Drug Interaction Probability Scale (5 points) and Naranjo (4 points), the drug-drug interaction between VRZ and ATRA was considered possible.^[18,19]^ We believe that the hypercalcemia and other clinical manifestations in the child were caused by the unique inhibition of ATRA metabolism by VRZ. In order to reduce the occurrence of such adverse effects, we recommend limiting the use of drugs that may inhibit CYP450 enzyme during ATRA treatment. Adverse reactions such as hypercalcemia should be closely monitored when there is a need for combination therapy in clinical practice. If necessary, salmon calcitonin, bisphosphonates, or other treatments for hypercalcemia can be added, or the dosage of ATRA can be reduced or even discontinued.

## 4. Summary and conclusion

In summary, we report a case of hypercalcemia caused by the interaction between ATRA and VRZ in a 14-year-old boy with APL and review the published literature. As scheduled, the child received maintenance chemotherapy with ATO, ATRA alone, and ATRA plus ATO, and VRZ for IFIs. No adverse reactions occurred during the concurrent use of ATO and VRZ. On the 12th day of the co-administration of ATRA and VRZ, the serum calcium level increased significantly and a series of accompanying symptoms occurred. Then VRZ was discontinued, ATRA was administered alone, and then ATRA combined with ATO was used as maintenance chemotherapy. The child’s serum calcium decreased and remained within the normal range. Through a literature review and exclusion of disease and concomitant medication factors, we believe that the hypercalcemia and other clinical manifestations may be caused by VRZ inhibiting the metabolism of ATRA.

Although it remains unclear whether hypercalcemia is caused by ATRA itself or a specific metabolite, and we were unable to obtain contemporaneous ATRA concentrations because of limitations. However, this case reminds us that ATRA is one of the important drugs for the treatment of APL, and CYP inhibitors including VRZ and other triazole antifungal drugs can enhance the effect of ATRA on calcium metabolism by inhibiting CYP450 enzyme. This underscores the importance of monitoring for adverse drug interactions and restricting the use of drugs that may inhibit CYP450 enzymes during ATRA treatment.

## Author contributions

**Writing – original draft:** Namei Wu.

**Writing – review & editing:** Namei Wu.

**Conceptualization and Supervision:** Zhihang Lin.

**Diagnosis and treatment:** Shuquan Zhuang.

**Drug concentration monitoring:** Shuifa Wu.

**Daily management and disease monitoring:** Zhiming Cai.

**Data and resource curation:** Xiaofang Wang.

## References

[1] Zhao J Liang JW Xue HL. The genetics and clinical characteristics of children morphologically diagnosed as acute promyelocytic leukemia. Leukemia. 2019;33:1387–99. 30575821 10.1038/s41375-018-0338-z

[2] Kutny MA Alonzo TA Abla O. Assessment of arsenic trioxide and all-trans retinoic acid for the treatment of pediatric acute promyelocytic leukemia: a report from the children’s oncology group AAML1331 trial. Jama Oncol. 2022;8:79–87. 34762093 10.1001/jamaoncol.2021.5206 PMC8587220

[3] Shariati A Moradabadi A Chegini Z Khoshbayan A Didehdar M. An overview of the management of the most important invasive fungal infections in patients with blood malignancies. Infect Drug Resist. 2020;13:2329–54. 32765009 10.2147/IDR.S254478 PMC7369308

[4] Pappas PG Kauffman CA Andes DR. Clinical practice guideline for the management of candidiasis: 2016 Update by the Infectious Diseases Society of America. Clin Infect Dis. 2016;62:e1–50. 26679628 10.1093/cid/civ933 PMC4725385

[5] Marill J Cresteil T Lanotte M Chabot GG. Identification of human cytochrome P450s involved in the formation of all-trans-retinoic acid principal metabolites. Mol Pharmacol. 2000;58:1341–8. 11093772 10.1124/mol.58.6.1341

[6] Hashmi Y Memon SF Khan YA Jabbar N Mansoor N. The effects of voriconazole on metabolism of all-trans retinoic acid in the treatment of acute promyelocytic leukemia: a case report. Cureus. 2021;13:e13337. 33747646 10.7759/cureus.13337 PMC7963433

[7] Sutherland J Kempton CL Curry MA. Continuation of all-trans retinoic acid despite the development of scrotal ulcerations in a Black male. J Oncol Pharm Pract. 2015;21:393–5. 24876163 10.1177/1078155214536244

[8] Buligon MP Mielke JC Chiesa J Ferrazzo KL. Rare labial ulcer related to the use of all-trans retinoic acid in a patient with acute promyelocytic leukemia. Spec Care Dentist. 2018;38:234–8. 29786869 10.1111/scd.12293

[9] Chen K Zhang X Ke X Du G Yang K Zhai S. Individualized medication of voriconazole: a practice guideline of the division of therapeutic drug monitoring, Chinese Pharmacological Society. Ther Drug Monit. 2018;40:663–74. 30192314 10.1097/FTD.0000000000000561 PMC6250289

[10] Kadam RS Van Den Anker JN. Pediatric clinical pharmacology of voriconazole: role of pharmacokinetic/pharmacodynamic modeling in pharmacotherapy. Clin Pharmacokinet. 2016;55:1031–43. 26979736 10.1007/s40262-016-0379-2

[11] Schwartz EL Hallam S Gallagher RE Wiernik PH. Inhibition of all-trans-retinoic acid metabolism by fluconazole in vitro and in patients with acute promyelocytic leukemia. Biochem Pharmacol. 1995;50:923–8. 7575674 10.1016/0006-2952(95)00213-j

[12] Vanier KL Mattiussi AJ Johnston DL. Interaction of all-trans-retinoic acid with fluconazole in acute promyelocytic leukemia. J Pediatr Hematol Oncol. 2003;25:403–4. 12759628 10.1097/00043426-200305000-00010

[13] Dixon KSP Hassoun AM. Pseudotumor cerebri due to the potentiation of all- trans retinoic acid by voriconazole. J Am Pharm Assoc. 2010;50:742–4. 10.1331/JAPhA.2010.09133 21071321

[14] Cordoba R Ramirez E Lei SH. Hypercalcemia due to an interaction of all-trans retinoic acid (ATRA) and itraconazole therapy for acute promyelocytic leukemia successfully treated with zoledronic acid. Eur J Clin Pharmacol. 2008;64:1031–2. 18584165 10.1007/s00228-008-0517-3

[15] Afacan Ozturk HB Albayrak M Maral S. Hypercalcemia associated with the interaction between all trans retinoic acid and posaconazole in an acute promyelocytic leukemia case. J Oncol Pharm Pract. 2021;27:2027–9. 33847196 10.1177/10781552211007889

[16] Bennett MT Sirrs S Yeung JK Smith CA. Hypercalcemia due to all trans retinoic acid in the treatment of acute promyelocytic leukemia potentiated by voriconazole. Leuk Lymphoma. 2005;46:1829–31. 16263588 10.1080/10428190500235298

[17] Niwa T Shiraga T Takagi A. Effect of antifungal drugs on cytochrome P450 (CYP) 2C9, CYP2C19, and CYP3A4 activities in human liver microsomes. Biol Pharm Bull. 2005;28:1805–8. 16141567 10.1248/bpb.28.1805

[18] Horn JR Hansten PD Chan LN. Proposal for a new tool to evaluate drug interaction cases. Ann Pharmacother. 2007;41:674–80. 17389673 10.1345/aph.1H423

[19] Naranjo CA Busto U Sellers EM. A method for estimating the probability of adverse drug reactions. Clin Pharmacol Ther. 1981;30:239–45. 7249508 10.1038/clpt.1981.154

